# Transcript and Protein Analysis Reveals Better Survival Skills of Monocyte-Derived Dendritic Cells Compared to Monocytes during Oxidative Stress

**DOI:** 10.1371/journal.pone.0043357

**Published:** 2012-08-15

**Authors:** Ilse Van Brussel, Dorien M. Schrijvers, Wim Martinet, Isabel Pintelon, Maartje Deschacht, Kathy Schnorbusch, Louis Maes, Johan M. Bosmans, Christiaan J. Vrints, Dirk Adriaensen, Paul Cos, Hidde Bult

**Affiliations:** 1 Laboratory of Cellular and Molecular Cardiology, University of Antwerp, Wilrijk, Antwerp, Belgium; 2 Laboratory of Physiopharmacology, University of Antwerp, Wilrijk, Antwerp, Belgium; 3 Laboratory of Cell Biology and Histology, University of Antwerp, Antwerp, Antwerp, Belgium; 4 Laboratory of Microbiology, Parasitology and Hygiene (LMPH), University of Antwerp, Wilrijk, Antwerp, Belgium; 5 Department of Cardiology, University Hospital of Antwerp, Edegem, Antwerp, Belgium; 6 Laboratory of Pharmacology, University of Antwerp, Wilrijk, Antwerp, Belgium; Statens Serum Institute, Denmark

## Abstract

**Background:**

Dendritic cells (DCs), professional antigen-presenting cells with the unique ability to initiate primary T-cell responses, are present in atherosclerotic lesions where they are exposed to oxidative stress that generates cytotoxic reactive oxygen species (ROS). A large body of evidence indicates that cell death is a major modulating factor of atherogenesis. We examined antioxidant defence systems of human monocyte-derived (mo)DCs and monocytes in response to oxidative stress.

**Methods:**

Oxidative stress was induced by addition of tertiary-butylhydroperoxide (*tert*-BHP, 30 min). Cellular responses were evaluated using flow cytometry and confocal live cell imaging (both using 5-(and-6)-chloromethyl-2,7-dichlorodihydrofluorescein diacetate, CM-H_2_DCFDA). Viability was assessed by the neutral red assay. Total RNA was extracted for a PCR profiler array. Five genes were selected for confirmation by Taqman gene expression assays, and by immunoblotting or immunohistochemistry for protein levels.

**Results:**

*Tert*-BHP increased CM-H_2_DCFDA fluorescence and caused cell death. Interestingly, all processes occurred more slowly in moDCs than in monocytes. The mRNA profiler array showed more than 2-fold differential expression of 32 oxidative stress–related genes in unstimulated moDCs, including peroxiredoxin-2 (PRDX2), an enzyme reducing hydrogen peroxide and lipid peroxides. PRDX2 upregulation was confirmed by Taqman assays, immunoblotting and immunohistochemistry. Silencing PRDX2 in moDCs by means of siRNA significantly increased CM-DCF fluorescence and cell death upon *tert*-BHP-stimulation.

**Conclusions:**

Our results indicate that moDCs exhibit higher intracellular antioxidant capacities, making them better equipped to resist oxidative stress than monocytes. Upregulation of PRDX2 is involved in the neutralization of ROS in moDCs. Taken together, this points to better survival skills of DCs in oxidative stress environments, such as atherosclerotic plaques.

## Introduction

Oxidative stress is believed to contribute to the development of several chronic human diseases, including atherosclerosis [1]–[5], and results from an imbalance between production of reactive oxygen species (ROS) and antioxidant defences [2]. Accumulating evidence has shown that ROS mediate innate immune reactions (direct killing of microbes by phagocytes) [6] and signal transduction from pathogen-recognition receptors [7].

Dendritic cells (DCs) are highly specialized antigen-presenting cells involved in both innate and adaptive immune responses. Under (oxidative) stress conditions, such as inflammation, infection and tissue damage, DCs rapidly take up foreign antigens to process and present them as soluble antigens in complexes with either class I or class II major histocompatibility complex (MHC) molecules [8]. We and others recently demonstrated that DC precursors, immature DCs and mature DCs accumulate in human atherosclerotic plaques [9]–[11], where they are exposed to oxidative stress [12], [13] and oxidized low density lipoproteins (oxLDL) [14], [15]. Therefore, it can be assumed that immature DCs will show phenotypic adaptations in order to function under oxidative stress situations. To investigate this hypothesis, monocytes and monocyte-derived (mo)DCs were exposed to a well known inducer of oxidative stress, namely tertiary-butylhydroperoxide (*tert*-BHP). *Tert*-BHP was selected since it acutely causes oxidative stress, resulting in cell toxicity [16]. Decomposition of *tert*-BHP to alkoxyl or peroxyl radicals accelerates lipid peroxidation chain reactions [17].

The effectiveness of the antioxidant defence systems was assessed by measuring intracellular ROS, which carries a real challenge as most ROS are highly reactive and very short-lived. We used 5-(and-6)-chloromethyl-2,7-dichlorodihydrofluorescein diacetate acetyl ester (CM-H_2_DCFDA) to evaluate intracellular oxidative stress, since it detects a very broad spectrum of ROS, including hydrogen peroxide (H_2_O_2_), alkoxyl radicals and peroxynitrite anions [18]. CM-DCF fluorescence was measured by flow cytometry (FCM), but as this technique cannot correct for potential differences in probe load between monocytes and moDCs, we also measured CM-DCF fluorescence in situ using confocal live cell imaging (LCI). LCI has the added advantage that cell scraping and isolation, which may induce different degrees of oxidative stress in both cell types, is avoided.

Secondly, survival of monocytes and moDCs was assessed by a neutral red viability assay.

To elucidate potential differences, we thirdly examined expression of oxidative stress-related genes (OSRGs) using a commercial PCR profiler array platform with 84 probe sets. Five genes with (1) direct pro-or antioxidant enzymatic activity, (2) more than 4-fold differential expression, and (3) a fairly high expression level in monocytes were selected for validation at the mRNA level (Taqman assays), and protein level (Western blot and immunohistochemistry).

Finally, we examined whether peroxiredoxin-2 (PRDX2), that showed strong upregulation in moDCs, was involved in the resistance of moDCs to *tert*-BHP-induced cell death. PRDX2 detoxifies H_2_O_2_ and alkyl hydroperoxides, two ROS which are mainly induced by *tert*-BHP. Hence, expression of PRDX2 in moDCs was silenced by using short-interfering RNA (siRNA).

## Materials and Methods

### Cell culture

Peripheral blood mononuclear cells were isolated by density gradient centrifugation from buffy coats (Antwerp blood transfusion centre, Red Cross Flanders). Subsequently, monocytes were purified by using anti-CD14-conjugated magnetic microbeads, as described [19]. One half of the CD14-positive monocytes was cultured overnight in RPMI supplemented with L-glutamine containing fungizone amphotericin (1 µg/mL) and gentamycin (10 µg/mL; all from Gibco, Invitrogen, Carlsbad, California, USA). The other half of CD14-positive cells was cultured for 6 days in RPMI supplemented with GM-CSF (5 ng/mL) and IL-4 (25 ng/mL) to obtain moDCs (both from Gentaur, Brussels, Belgium). For LCI experiments, poly-D-lysine coated glass bottom culture dishes (MatTek, Ashland, USA) were used; for all other experiments, cells were cultured in polystyrene 24-well plates (Corning Inc, Amsterdam, The Netherlands). Evaluation of cell viability was based on the incorporation of the supravital dye neutral red as described [20]. All cell cultures were performed at 37°C in a humidified atmosphere supplemented with 5% CO_2_.

### CM-DCF fluorescence measured by FCM

**Figure 1:**
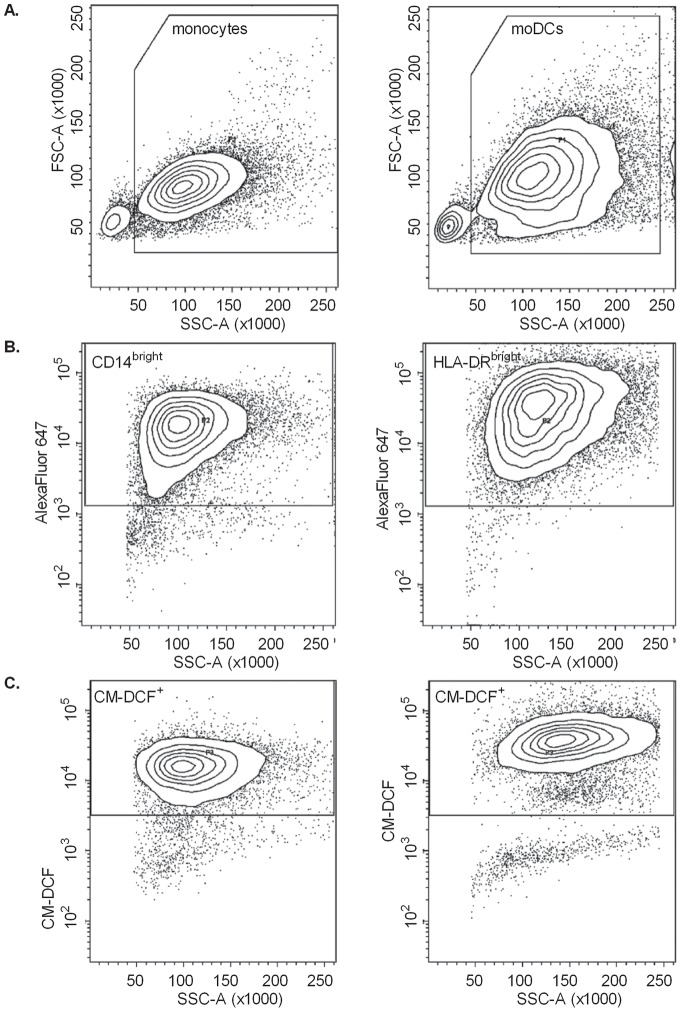
Gating strategy to evaluate ROS content, measured as CM-DCF fluorescence, in monocytes and moDCs by flow cytometry (FCM). **A.** First, monocyte or moDC populations were distinguished from cellular debris. **B.** From the gated populations, CD14^bright^ or HLA-DR^bright^ cells were selected. C. Only in this bright fluorescent population, CM-DCF fluorescence was evaluated in FL-1.

After harvesting and washing, cells were labelled with Alexa Fluor 647-conjugated anti-human CD14 (monocytes) or anti-human HLA-DR (moDCs), both from Biolegend (Uithoorn, The Netherlands). After 25 min, excess antibody was removed by centrifugation (6 min, 450×g) and cells were suspended in PBS with 10 µM CM-H_2_DCFDA (Molecular Probes, Invitrogen, Merelbeke, Belgium). After 20 min, extracellular probe was removed by centrifugation (6 min, 450×g), and cells were suspended in 100 µl PBS. CM-DCF fluorescence was measured immediately or after incubation (30 min, 37°C) with 200 µM *tert*-BHP (Sigma, Bornem, Belgium) on a FacsCanto II flow cytometer (BD, Erembodegem, Belgium) using automatic compensation settings, and 20,000 events were analyzed with FacsDiva 6.1 software. CM-DCF fluorescence was evaluated within the population of CD14^bright^ cells for monocytes and HLA-DR^bright^ cells for moDCs (figure 1).

### CM-DCF fluorescence measured by confocal LCI

**Figure 2:**
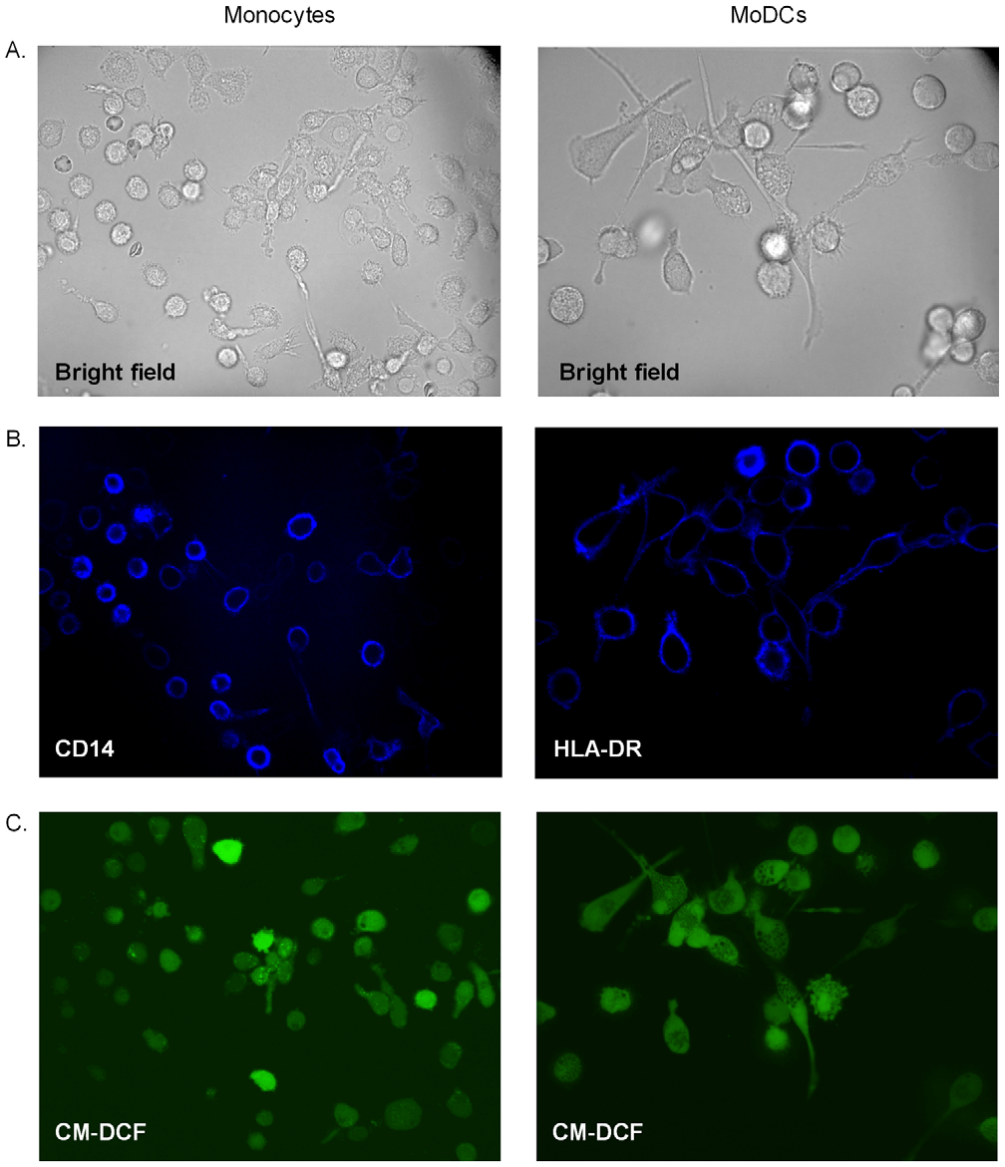
Evaluation of ROS content, measured as CM-DCF fluorescence, in monocytes and moDCs by confocal LCI. **A.** Bright field snapshots and **B.** CD14 or HLA-DR fluorescence were used to select undifferentiated monocytes (CD14^bright^) and well differentiated moDCs (HLA-DR^bright^). **C.** Cells with bright fluorescence were selected as regions of interest (ROIs) and mean fluorescence intensities within every ROI were measured at 2 min intervals; mean fluorescence of each ROI was automatically calculated by the Volocity 5.4.3 software.

**Figure 3:**
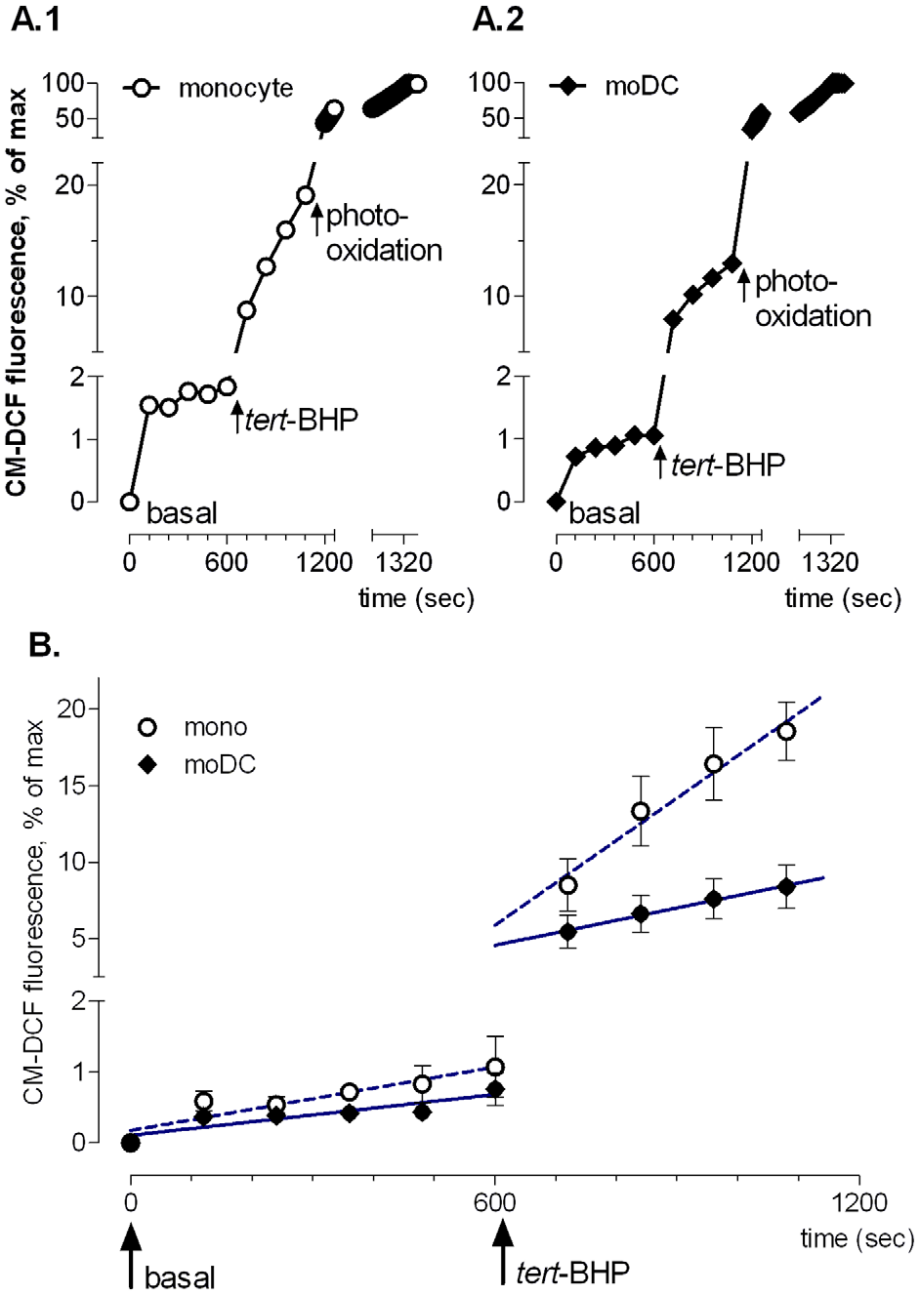
CM-DCF fluorescence measured by confocal LCI. **A.** *Time course of CM-DCF measurement by LCI.* ROS content, measured as CM-DCF fluorescence, was evaluated before (0–600 sec) and after (600–1200 sec) addition of *tert*-BHP. An immediate steep increase of CM-DCF fluorescence was observed after exposure to 200 µM *tert*-BHP at 600 sec, followed by a slower linear rise between 720–1200 sec. After 1200 sec, photo-oxidation of remaining CM-DCFH through continuous illumination was used to obtain maximum fluorescence. Representative examples of mean fluorescence of a single monocyte (A.1) and a monocyte-derived DC (A.2). Data were corrected for probe load as follows: [(fluorescence at time x – fluorescence at time zero)/(maximum fluorescence – fluorescence at time zero)] x 100. **B.** *Slower increase of tert-BHP-induced CM-DCF fluorescence in monocyte-derived DCs compared to monocytes.* Results are expressed as percent increase per 10^3^ sec and show mean ± SEM of four experiments, each based on the average of 12–15 cells, and linear regression lines calculated for basal fluorescence (0 to 600 sec) and the slow rise after *tert*-BHP addition (720 to 1200 sec).

Culture medium was removed and replaced by 2 mL PBS with Alexa Fluor 647-conjugated anti-human CD14 (monocytes) or anti-human HLA-DR (moDCs). After 25 min, cells were rinsed with PBS, and 10 µM CM-H_2_DCFDA in PBS was added. After 20 min, the dish was placed on a Zeiss Axiovert 200 microscope attached to an Ultra*VIEW* spinning confocal Live Cell Imager (Perkin Elmer, Seer Green, UK). After taking a bright field snapshot (figure 2A), red light (excitation 647 nm) was used to visualize HLA-DR or CD14 fluorescence (figure 2B). Then, CM-DCF fluorescence was imaged for 18 min by repeated exposure to blue light (excitation 488 nm; 500 msec exposure time) at 2 min intervals to minimize exposure to laser light (figure 2C). First, basal CM-DCF fluorescence was studied for 10 min at 25°C, then 200 µM *tert*-BHP was added and fluorescence was recorded for another 8 min. Thereafter, cells were continuously illuminated during 4 min to determine the individual probe load of each cell by causing photo-oxidation of remaining 5-(and-6)-chloromethyl-2,7-dichlorofluorescein and 2 images per second were recorded (figure 3A). Maximum CM-DCF fluorescence provided a measure of total CM-DCF content per cell.

Snapshots of CD14 or HLA-DR fluorescence were used to select 12–15 undifferentiated monocytes (CD14^bright^) or moDCs (HLA-DR^bright^) as “regions of interest" (ROIs, figure 2C). The mean CM-DCF fluorescence of every ROI was calculated for each time point using Volocity 5.4.3. To correct for probe load, data of each ROI were normalized as follows: [(fluorescence at a given time – initial fluorescence)/(maximum fluorescence – initial fluorescence)] ×100. Finally, the average of the 12–15 ROIs in each well was calculated and used for further analysis.

### PCR profiler array

RNA of 5.0×10^5^ monocytes or moDCs from four donors was extracted using the Absolutely RNA Microprep Kit (Stratagene, La Jolla, California, USA). One part of the RNA extracts was pooled for the PCR profiler array, the other part was stored at −80°C for confirmation by means of Taqman gene expression assays. cDNA was prepared with the RT^2^ First Strand Kit (SABiosciences, Frederick, Maryland, USA). A PCR profiler array specific for 84 OSRGs was performed (RT^2^ SYBR Green/ROX qPCR Master Mix; SABiosciences) in 96-well microtiter plates on an ABI 7300 instrument (Applied Biosystems, California, USA). The plates were incubated for 10 min at 95°C, followed by 40 cycles of denaturation at 95°C for 15 sec and annealing/extension at 60°C for 60 sec. After the PCR program, a melting curve program was run and the first derivative dissociation curve was generated.

### PCR profiler array analysis: ΔΔCt method

**Figure 4:**
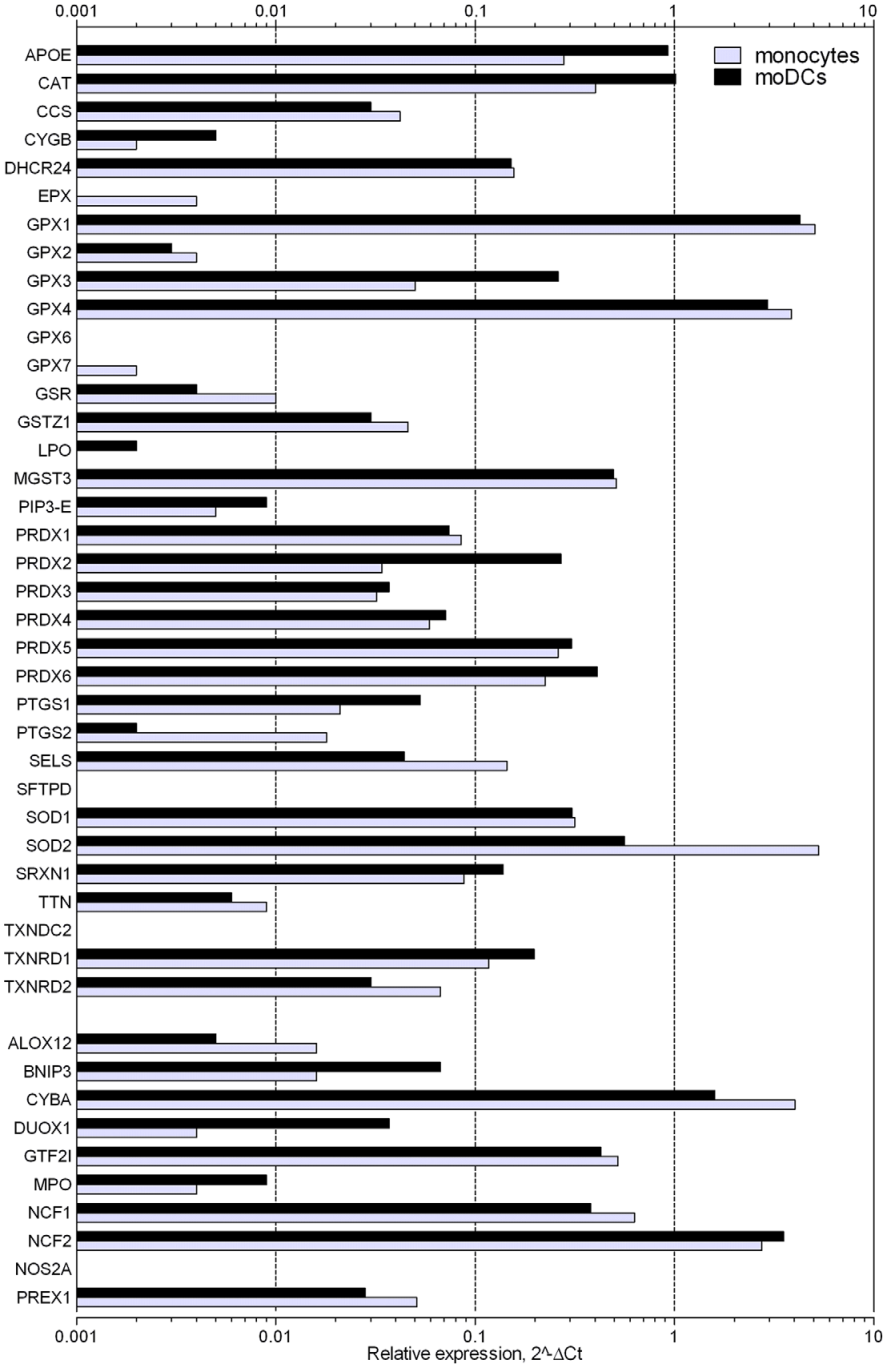
Expression of genes with pro-or antioxidant properties in monocytes and moDCs relative to expression of reference genes (ACTB and HPRT1) reported as fold difference (2−ΔCt). The relative mRNA expression of 10 pro-oxidants is shown at the bottom (ALOX12 to PREX1), all other genes (ApoE to TXNRD2) exert antioxidant effects.

The ΔΔCt method was applied according to the PCR profiler array manual. Of the five reference genes (HKGs) included in the PCR profiler array (β2 microglobulin (B2M), HPRT1, ribosomal protein L13a (RPL13A), glyseraldehyde-3-phosphate dehydrogenase (GAPDH), ACTB), only expression of HPRT1 and ACTB was not different between both cell types (P>0.05), and therefore the average of these HKGs was used for further analysis. Then, ΔCt of the gene of interest (GOI) was calculated: ΔCt  =  Ct^GOI^−Ct^[(HPRT1+ACTB)/2]^. To determine the expression level of the GOI in monocytes and moDCs, 2^−ΔCt^ was calculated, which gives expression relative to HKGs as fold difference. The values are presented in figure 4 for monocytes and moDCs.

To investigate changes in mRNA expression during differentiation of monocytes to moDCs, we then used monocytes as control group, and unstimulated moDCs as experimental group, and calculated ΔΔCt: ΔΔCt  =  ΔCt^(experimental group)^−ΔCt^(control group)^. The fold change from monocytes to moDCs was calculated as 2^−ΔΔCt^. If fold change was greater than 1, the result was considered as fold-upregulation. If fold change was less than 1, the negative inverse of the result was considered as fold-downregulation. These values (2^−ΔΔCt^) are presented in table 1 for monocytes ( =  control group, set to 1) and moDCs.

**Table 1:** PCR profiling array analysis of oxidative stress related genes (OSRG) with two-fold or more up-or downregulated expression during differentiation of monocytes(mono) into monocyte-derived DCs (moDC).

Description	Symbol	Expression in monocytes set to 1	Fold up-or downregulation in moDCs
**Upregulated in DC**			
Apolipoprotein E	APOE*	1	3.32
BCL2/adenovirus E1B 19kDa interacting protein 3	BNIP3	1	4.06
Catalase	CAT*	1	2.53
Chemokine (C-C motif) ligand 5	CCL5	1	44.02
Cytoglobin	CYGB	1	2.08
Dual oxidase 1	DUOX1	1	9.25
Epoxide hydrolase 2, cytoplasmic	EPHX2	1	4.79
Glutathione peroxidase 3 (plasma)	GPX3	1	5.17
Myeloperoxidase	MPO	1	2.31
Peroxiredoxin 2	PRDX2	1	8.00
Prion protein (p27-30)	PRNP	1	5.86
Prostaglandin-endoperoxide synthase 1	PTGS1	1	2.55
Serum/glucocorticoid regulated kinase 2	SGK2	1	17.51
Thioredoxin domain-containing 2	TXNDC2	1	2.17
**Downregulated in DC**			
Arachidonate 12-lipoxygenase	ALOX12	1	−3.23
Angiopoietin-like 7	ANGPTL7	1	−3.45
Cytochrome b-245, alpha polypeptide	CYBA*	1	−2.50
Dual specificity phosphatase 1	DUSP1	1	−2.86
Eosinophil peroxidase	EPX	1	−4.17
Glutathione peroxidase 7	GPX7	1	−50.0
Glutathione reductase	GSR*	1	−2.38
Keratin 1 (epidermolytic hyperkeratosis)	KRT1	1	−4.35
Metallothionein-like 5, testis-specific	MTL5	1	−4.55
PDZ and LIM domain 1 (elfin)	PDLIM1	1	−3.13
Polynucleotide kinase 3′-phosphatase	PNKP	1	−2.38
Prostaglandin-endoperoxide synthase 2	PTGS2	1	−11.1
Selenoprotein S	SELS	1	−3.23
Selenoprotein P, plasma, 1	SEPP1	1	−2.56
Surfactant, pulmonary-associated protein D	SFTPD	1	−5.26
Sirtuin	SIRT2	1	−2.33
Superoxide dismutase 2, mitochondrial	SOD2*	1	−9.09
Thioredoxin reductase 2	TXNRD2	1	−2.22

Fold expression with monocytes as control group (expression set to 1) and unstimulated moDCs as the experimental group (see section ‘materials and methods’).

* also documented by Le Naour [28].

### Taqman gene expression assay

RNA samples were converted to cDNA with reverse transcriptase from Eurogentec (Seraing, Belgium). Homo sapiens-specific Taqman gene expression assays (Applied biosystems, California, USA) were purchased for dual oxidase (DUOX)-1 (Hs00213694_m1), superoxide dismutase 2 (SOD2, Hs00167309_m1), glutathione peroxidise-3 (GPX3) (Hs00173566_m1), GPX7 (Hs00210410_m1), PRDX2 (Hs03044902_g1), as well as for an endogenous control ACTB (Hs99999903_m1). Real-time TaqMan qPCR was performed using the ABI 7300 Sequence Detection System and 96-well plates using the same reaction conditions as described for the PCR profiler array (vide supra). Target and endogenous control gene (ACTB) reactions were run in duplicate. Data were analyzed with the ΔΔCt method with all target gene reaction results normalized to those of the endogenous control gene ACTB to correct for any differences in efficiency of the reverse transcription reactions.

### Western blot

Cells (2–10×10^6^) were prepared for Western blot analysis as described previously [20]. In brief, they were lysed in 1 mL Laemmli-buffer, heat-denaturated for 5 min in a boiling water bath and loaded on a 4–12% Bis-Tris gel (Invitrogen, Carlsbad, CA, USA). Then, proteins were transferred to Immobilon-P membranes (Millipore, Billerica, MA, USA), which were blocked using 5% non-fat dry milk in Blotting (transfer) buffer, and incubated overnight at 4°C with primary antibodies (mouse anti-SOD2, mouse anti-PRDX2, goat anti-GPX3, all from R&D Systems, Abingdon, UK; mouse anti-GPX7, Thermo Scientific Pierce Antibodies, Waltham, Massachusetts, USA; mouse anti-DUOX1, Novus Biologicals, Cambridge, UK; and mouse anti-beta-actin (clone AC15), Sigma, St. Louis, Missouri, USA), followed by 1 h incubation at RT with a secondary horseradish peroxidase (HRP)-conjugated antibody (Dako, Glostrup, Denmark). Bands were visualized via chemiluminescence (SuperSignal West Femto Maximum Sensitivity Substrate, Pierce, Waltham, Massachusetts, USA) using a Lumi-Imager (Roche, Mannheim, Germany).

### Immunohistochemistry

Cells were harvested, dried on adhesive microscope slides (37°C) for 2 h, fixed with 4% paraformaldehyde (10 min) and washed in Tris-saline buffer (TBS) for 5 min. Cells were blocked with 2% horse serum for 20 min and then incubated overnight with primary antibodies (monoclonal anti-human SOD2 antibody, 1/100; monoclonal anti-human PRDX2 antibody, 1/500; monoclonal anti-human GPX7 antibody, 1/1000; monoclonal anti-human DUOX1 antibody, 1/2000; affinity-purified goat anti-human GPX3 antibody, 1/1000). Endogenous peroxidase activity was blocked using methanol/distilled water (v/v 1/1) and 0.3% H_2_O_2_ for 20 min. After washing with TBS, cells were incubated for 30 min with species-specific biotinylated secondary antibody and washed again twice in TBS. Finally, cells were incubated for 1 h in ABC-peroxidase solution, rinsed three times in TBS and stained with 3-amino-9-ethylcarbazole (AEC).

### siRNA mediated gene silencing

Cells were transfected with 50 nM ON-TARGETplus SMARTpool siRNA specific to PRDX2 (Dharmacon, Lafayette, Colorado, USA) using HiPerfect transfection reagent (Qiagen, Valencia, CA, USA) according to the instructions of the manufacturer. ON-TARGETplus Non-Targeting Pool siRNA (Dharmacon) was used as a negative control. Silencing efficiency was monitored after 24 h by qPCR and after 48 h by Western blot.

### Statistical analysis

Variables that failed normality or showed heterogeneity of variances were logarithmically transformed. In viability experiments, one sample t-test was used for comparison to unstimulated cells (fixed value of 100%). For LCI experiments, the increase in CM-DCF fluorescence before and after addition of *tert*-BHP was determined with linear regression analysis. Repeated measures ANOVA with *differentiation* and *stimulus* as within-subject factors combined with Dunnett's post-hoc test were used to analyze differences between cell types and stimuli. Differences between non-transfected moDCs and moDCs transfected with PRDX2-targeting siRNA were analyzed with the paired Student's t-test. P<0.05 was considered statistically significant. Data are shown as mean ± SEM, n represents the number of independent experiments (i.e. buffy coats).

## Results

### Oxidative stress induced less ROS production and cell death in moDCs, compared to monocytes

**Figure 5:**
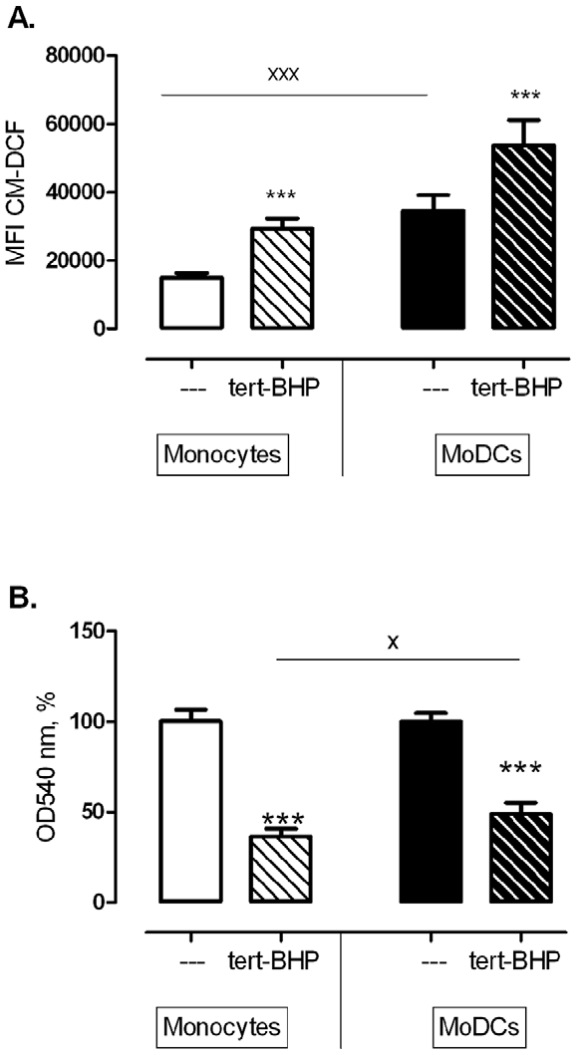
Tert-BHP induced ROS production and cell death in monocytes and moDCs. **A.** *CM-H_2_DCF fluorescence measured by FCM, before and after addition of tert-BHP (200* *µM).* Repeated measures ANOVA (with *differentiation* and *stimulus* as within-subject factors), revealed a significant increase after *tert*-BHP and significantly more fluorescence in moDCs than in monocytes, but both cell types responded similar to *tert*-BHP, as indicated by the lack of interaction (n = 8). **B.** *Cell death induced in monocytes and moDCs by tert-BHP (200* *µM, 30* *min).* T*ert*-BHP induced cell death in both cell types, but moDCs appeared to be more resistant to *tert*-BHP induced cell death. ***P<0.001 tert-BHP versus untreated control (One sample t-test, n = 16), ^x^P<0.05, ^xxx^P<0.001 monocytes versus moDCs (Repeated measures ANOVA, n = 16).

FCM showed that CM-DCF fluorescence was higher in moDCs, compared to monocytes (P = 0.0032). Addition of *tert*-BHP significantly increased CM-DCF fluorescence in both CD14^bright^ monocytes and HLA-DR^bright^ moDCs (P<0.0001), and this occurred to the same extent in both cell types, as indicated by the lack of interaction in the repeated measures ANOVA (P = 0.2945; figure 5A).

LCI, which had the advantages that it was possible to analyze adherent cells, and to correct for differences in probe load between both cell types (figures 2 and 3A), demonstrated that basal CM-DCF fluorescence increased gradually in CD14^bright^ monocytes and HLA-DR^bright^ moDCs. Since the slopes were not different (P = 0.235), it was possible to calculate a pooled slope (1.2±0.2), and it then appeared that the curve of the monocytes was more elevated than that of moDCs (P = 0.025), reflecting increased ROS levels. *tert*-BHP stimulation caused a biphasic response: an immediate, sharp rise of CM-DCF fluorescence that was not significantly different between monocytes and moDCs (P = 0.2143) was followed by a protracted, linear increase in time. However, in the linear phase the slopes of the regression lines of CM-DCF fluorescence were different between cell types (*monocytes*: 27.7±7.3; *moDCs*: 8.2±4.3% increase/10^3^ sec); P = 0.029; figure 3B): the rise of ROS occurred about 3-fold slower in moDCs than in monocytes.

With regard to the sensitivity to *tert*-BHP-induced cell death, neutral red assays revealed that *tert*-BHP induced cell death in monocytes and moDCs (P<0.001), but moDCs were significantly more resistant to cell death (P<0.001; figure 5B).

### mRNA expression of pro- and antioxidant genes in moDCs

**Figure 6:**
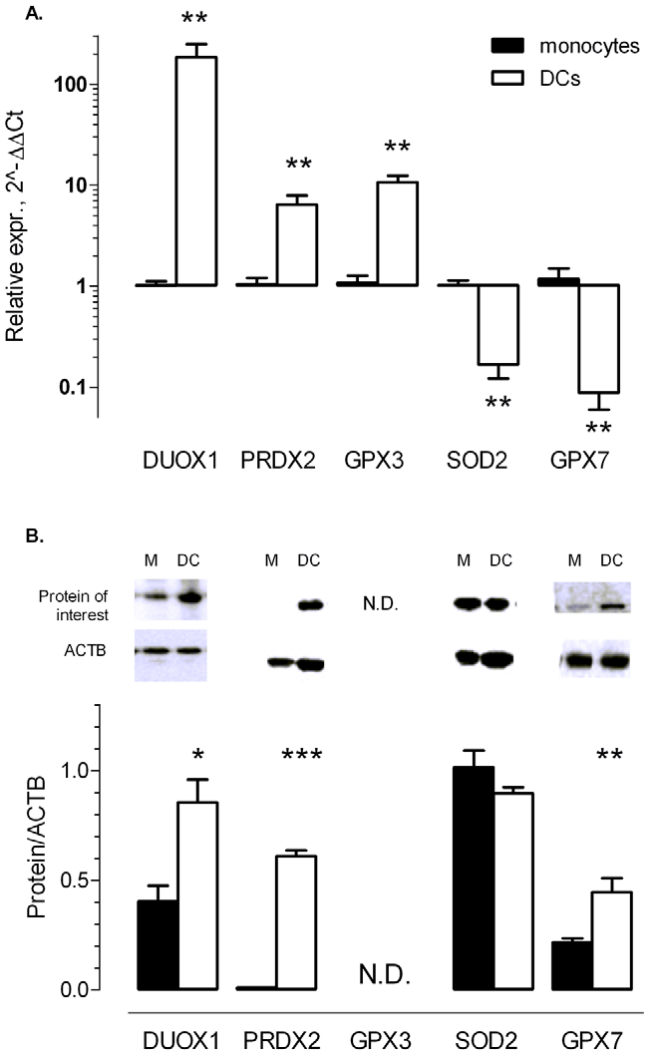
mRNA and protein expression of pro-and antioxidants in moDCs and monocytes. A. *Relative mRNA expression of DUOX1, PRDX2, GPX3, SOD2 and GPX7 in monocytes and moDCs*. mRNA expression was analyzed using Taqman assays in individual non-pooled RNA extracts. Data were normalized to expression of ACTB and monocytes served as control group (expression set to 1). **B.** *Western blot analysis of DUOX1, PRDX2, SOD2 and GPX7* during differentiation from monocytes (M) to moDCs (DC). The antibody against GPX3 did not yield signals that were distinguishable from background noise in the immunoblots. Representative blots (top panels) and mean densitometric ratios of protein of interest/ACTB. Values show mean ± SEM, n = 4. *N.D.  =  not detectable.* *P<0.05, **P<0.01, ***P<0.001.

Sixty-four (out of 84) OSRG transcripts (76%) were detectable (Ct<35) via a PCR profiler array in monocytes and moDCs. The 44 genes with known pro-or antioxidant properties are shown in figure 4, while genes showing a ≥2.0-fold expression difference are summarized in table 1. Fourteen genes were upregulated in moDCs versus monocytes, whereas 18 genes were downregulated. Five genes showing more than 4-fold differential expression were selected for validation at mRNA and protein level: DUOX1, a member of the peroxiredoxin family PRDX2, the glutathione peroxidases GPX3 and GPX7, and SOD2. For those genes, Taqman assays confirmed the altered gene expression in moDCs, as compared to monocytes (figure 6A). Moreover, the changes were very consistent in all donors tested, though the expression of GPX7 was rather low in monocytes and showed more variability among donors.

### Protein expression of pro-and antioxidants in moDCs

Western blot analysis revealed increased expression of DUOX1 and PRDX2 during differentiation of monocytes to moDCs (figure 6B), confirming mRNA results. SOD2 and GPX7 were downregulated at the mRNA level during DC differentiation, but SOD2 protein was not differentially expressed and even increased GPX7 protein expression was observed in moDCs (figure 6B). The antibody against GPX3 did not yield signals that were distinguishable from background noise in the immunoblots. All results from Western blots could be confirmed by immunohistochemistry. In addition, for GPX3, immunohistochemistry showed that moDCs stained far more positive than monocytes, in accordance with the mRNA results (data not shown).

### Lipid-mediated siRNA transfection to suppress PRDX2 expression in moDCs

**Figure 7:**
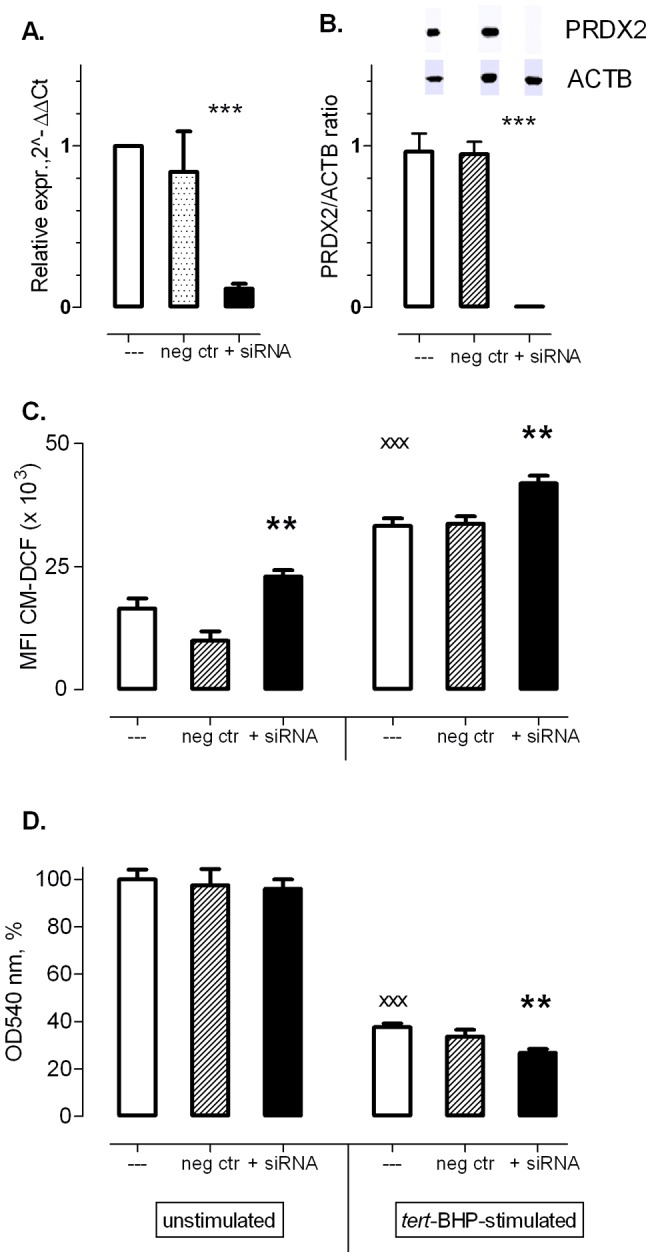
Suppression of PRDX2 mRNA and protein expression in moDCs by means of lipid-mediated siRNA transfection leads to enhanced sensitivity of moDCs to tert-BHP. **A.** Reduced PRDX2 mRNA levels in unstimulated moDCs after transfection with anti-PRDX2 siRNA [qPCR; 2^−ΔΔCt^ normalization using ACTB as reference gene and the non-transfected sample (---) as control (set to 1, n = 4)]. **B.** Reduction of PRDX2 protein in unstimulated moDCs monitored by Western Blot. Data were normalized using ACTB as reference gene (n = 4). **C.** CM-DCF fluorescence was used as index for intracellular ROS levels in non-transfected moDCs and moDCs transfected with PRDX2-targeting siRNA, with or without tert-BHP stimulation (200 μM, 30 min, n = 8). **D.** Neutral Red assay was used as index viability index of non-transfected moDCs and moDCs transfected with PRDX2-targeting siRNA, with or without tert-BHP stimulation (200 μM, 30 min, n = 8). **P<0.01 vs. untransfected (--) and negative control (neg. ctr), ^xxx^P<0.001 vs. Unstimulated, paired Student's t-test (two-tailed).

Because peroxiredoxins are involved in the reduction of H_2_O_2_ and lipid peroxides, we investigated whether the resistance of moDCs to *tert*-BHP-induced cell death was a direct result of the high upregulation of intracellular PRDX2 expression. Therefore, expression of PRDX2 in moDCs was suppressed by siRNA (figures 7A, 7B), leading to silencing at mRNA (89±3% at 24 h) and protein level (100% at 48 h). Silencing of PRDX2 expression significantly increased CM-DCF fluorescence in unstimulated moDCs (P = 0.039) and in *tert*-BHP-stimulated samples (P = 0.050; figure 7C). Moreover, PRDX2 silencing significantly raised the sensitivity of moDCs to *tert*-BHP-induced cell death (P = 0.008; figure 7D).

## Discussion

Oxidative stress has been shown to inhibit the capacity of antigen-presenting cells to process antigens and to initiate a primary T-cell response [21]. Antigen-presenting cells such as DCs, are exposed to oxidative stress at inflammatory sites (e.g. atherosclerotic plaques). Therefore, they might show phenotypic alterations to be able to function in oxidative stress environments. The present study documented for the first time differential responses of moDCs and monocytes to oxidative stress induced by *tert*-BHP. LCI experiments showed clearly that moDCs were better capable of neutralizing *tert*-BHP-induced ROS than monocytes. FCM appeared to be less appropriate to compare responses between *different cell types* to the stimulus – due to differences in probe load, morphology, adherence and other variations between both cell types (figure 1). Yet, it is very suitable to investigate whether or not a certain stimulus induces ROS in one specific cell type. It should be mentioned that the reliability of all probes that are currently used to monitor ROS has been questioned with respect to specificity, cell retention and auto bleaching, among others [22]. We selected CM-H_2_DCFDA, an improved version of the original dye (H_2_DCF), to obtain a better retention of the dye in living cells, because of the added chloromethyl group [23]. Moreover, with regard to photosensitivity [24], the use of a spinning disk confocal microscope minimizes photo-oxidation during the real-time recording of CM-H_2_DCFDA, by ensuring that each point in the sample is subjected to a limited dose of radiation and by the high speed acquisition of the spinning disk [25]–[27].

Next, the viability assay gave direct support to the hypothesis that moDCs are better equipped to survive in highly oxidative environments. *Tert*-BHP induced a significant and rapid cell death in both cell types, but moDCs were more resistant to *tert*-BHP-induced cell death than monocytes.

To identify antioxidant enzymes that could be responsible for the resistance of moDCs to *tert*-BHP, a PCR profiler array specific for oxidative stress and antioxidant-related pathways was used. The array documented differential expression (2-fold or more) for 32 out of 64 detectable genes. From these 32 genes, upregulation of catalase and apolipoprotein E (APOE), and downregulation of cytochrome b-245 alpha polypeptide (CYBA), glutathione reductase (GSR) and SOD2 in moDCs was previously detected in a random oligonucleotide microarray study of 6,300 genes [28]. In addition to those five genes (CYBA, SOD2, GSR, APOE, catalase) which showed high mRNA expression levels in monocytes (>0.01; figure 4), the profiler array uncovered differential expression of another 27 genes that were less abundantly expressed. Expression of GPX3 and PRDX2, which are genes encoding enzymes that can detoxify H_2_O_2_ and lipid hydroperoxides [29], [30], and expression of myeloperoxidase (MPO), which uses H_2_O_2_ as a substrate with chloride as a cosubstrate to form hypochlorous acid [31], were increased in moDCs. Beside MPO, another gene involved in ROS generation, namely DUOX1, was upregulated during moDC differentiation. This gene encodes a protein of the NOX/DUOX family of NADPH oxidases, that has the regulated production of ROS as its main function [32]. It has already been described that DUOX1 is induced in response to IL-4 and IL-13 [32]. As IL-4 was used to differentiate monocytes into moDCs, this presumably explains the upregulation of DUOX1 in moDCs. Nonetheless, the ROS induced by those pro-oxidants (MPO and DUOX1) presumably contribute to the oxidative stress in moDCs. On the other hand, expression of two peroxidases, eosinophil peroxidase and GPX7, was downregulated in moDCs at the mRNA level. However, their expression at the mRNA level (0.004, 0.002 relative to ACTB and HPRT1) was very low in comparison to GPX3 (0.05), catalase (0.5) or PRDX2 (0.03) (figure 4).

Immunoblotting or immunohistochemistry showed that the upregulated transcription of PRDX2, GPX3 and DUOX1 was translated in a two-fold increase at the protein level. Remarkably, for genes showing downregulated mRNA levels in moDCs, protein expression did not differ between both cell types (SOD2), or even displayed increased expression in moDCs (GPX7). For SOD2, this was also reported by Le Naour et al. [28]. SOD2, which is a mitochondrial protein, might be protected from proteolytic degradation by its localization. Moreover, moDCs are known to contain a larger number of mitochondria than monocytes [33]. The reason for the poor correlation between decreased mRNA and upregulated protein levels of GPX7 remains unknown. Whatever the reason is, even the two antioxidants that were downregulated at mRNA level, showed unaltered or even increased protein expression. Taken together, these protein data suggest that in general, moDCs exhibit higher intracellular antioxidant capacities in comparison to monocytes, which is in accordance with two other studies [34], [35].

Finally, we evaluated whether the high expression of PRDX2 protein in moDCs, which was undetectable in monocytes, contributed to the resistance of moDCs to *tert*-BHP-induced cell death. PRDX2 detoxifies H_2_O_2,_ lipid peroxides and other toxic organic peroxides, that are formed upon *tert*-BHP exposure. Moreover, it is also more efficient in neutralizing H_2_O_2_ than catalase or glutathione peroxidase [36], [37]. Therefore, expression of PRDX2 was silenced by introducing siRNA in moDCs, resulting in robust suppression of mRNA and complete suppression of protein levels.

After PRDX2 knock down CM-DCF fluorescence was significantly higher in moDCs, before and after *tert*-BHP exposure. Moreover, *tert*-BHP caused significantly more cell death when PRDX2 expression was suppressed. These findings indicate that PRDX2 in moDCs is indeed an important factor in the neutralization of ROS induced by *tert*-BHP, leading to increased survival.

### Conclusions

The data of the present study provide strong evidence that moDCs are better equipped to respond to induced oxidative stress in comparison with monocytes, and this presumably explains why they show less cell death in highly oxidative environments. Both mRNA and protein analysis revealed that moDCs exhibit higher intracellular antioxidant capacities. The present study is also the first to show that PRDX2 is involved in the neutralization of *tert*-BHP-induced ROS within moDCs. Not only the intracellular ROS content of moDCs increased when PRDX2 expression was knocked down, but also the sensitivity of moDCs to *tert*-BHP-induced cell death was significantly enhanced. Taken together, this points to better survival skills of DCs in oxidative stress environments, such as atherosclerotic plaques.
